# Characterization and analysis of the *Burkholderia pseudomallei* BsaN virulence regulon

**DOI:** 10.1186/s12866-014-0206-6

**Published:** 2014-08-01

**Authors:** Yahua Chen, Imke Schröder, Christopher T French, Artur Jaroszewicz, Xiao Jie Yee, Boon-Eng Teh, Isabelle J Toesca, Jeff F Miller, Yunn-Hwen Gan

**Affiliations:** 1 Department of Biochemistry, Yong Loo Lin School of Medicine, National University of Singapore, Singapore 117597, Singapore; 2 Department of Microbiology, Immunology and Molecular Genetics, The University of California Los Angeles, Los Angeles 90095, CA, USA; 3 Department of Molecular, Cell and Developmental Biology, The University of California Los Angeles, Los Angeles 90095, CA, USA; 4 California NanoSystems Institute, The University of California Los Angeles, Los Angeles 90095, CA, USA; 5 Molecular Biology Institute, The University of California Los Angeles, Los Angeles 90095, CA, USA; 6 Immunology Program, Yong Loo Lin School of Medicine, National University of Singapore, Singapore 117597, Singapore

## Abstract

**Background:**

*Burkholderia pseudomallei* is a facultative intracellular pathogen and the causative agent of melioidosis. A conserved type III secretion system (T3SS3) and type VI secretion system (T6SS1) are critical for intracellular survival and growth. The T3SS3 and T6SS1 genes are coordinately and hierarchically regulated by a TetR-type regulator, BspR. A central transcriptional regulator of the BspR regulatory cascade, BsaN, activates a subset of T3SS3 and T6SS1 loci.

**Results:**

To elucidate the scope of the BsaN regulon, we used RNAseq analysis to compare the transcriptomes of wild-type *B. pseudomallei* KHW and a *bsaN* deletion mutant. The 60 genes positively-regulated by BsaN include those that we had previously identified in addition to a polyketide biosynthesis locus and genes involved in amino acid biosynthesis. BsaN was also found to repress the transcription of 51 genes including flagellar motility loci and those encoding components of the T3SS3 apparatus. Using a promoter-*lacZ* fusion assay in *E. coli*, we show that BsaN together with the chaperone BicA directly control the expression of the T3SS3 translocon, effector and associated regulatory genes that are organized into at least five operons (*BPSS1516-BPSS1552*). Using a mutagenesis approach, a consensus regulatory motif in the promoter regions of BsaN-regulated genes was shown to be essential for transcriptional activation.

**Conclusions:**

BsaN/BicA functions as a central regulator of key virulence clusters in *B. pseudomallei* within a more extensive network of genetic regulation. We propose that BsaN/BicA controls a gene expression program that facilitates the adaption and intracellular survival of the pathogen within eukaryotic hosts.

## Background

Melioidosis is a serious and often fatal infectious disease common to Southeast Asia and Northern Australia caused by the Gram-negative soil bacterium *Burkholderia pseudomallei. B. pseudomallei* is a highly versatile pathogen capable of surviving inside mammalian cells and in many environmental niches. The bacterium can infect numerous animal species, amoebae, nematodes, and tomato plants [1]–[5], and has been previously found within the tissues of exotic grasses in Australia [6]. The environmental origin of *B. pseudomallei* and its promiscuous host range have shaped the hypothesis that some of its genetic loci evolved in the rhizosphere as anti-predation determinants that subsequently promote “accidental” virulence in humans and animals. In recent years, important advances have been made in understanding the pathogenic mechanisms of *B. pseudomallei* including the roles of the Type III and Type VI Secretion Systems (T3SS, T6SS) [7]–[11]. *B. pseudomallei* contains three T3SSs and six T6SSs, but only T3SS3 (also referred to as the *Burkholderia* secretion apparatus, or T3SS_Bsa_) and T6SS1 are critical for pathogenesis in mice and hamsters [7],[12],[13].

Expression of the T3SS3 and T6SS1 gene clusters is tightly controlled, both temporally and spatially, during the *B. pseudomallei* intracellular lifecycle. We have identified a regulatory cascade that coordinately activates T3SS3 and T6SS1 gene expression in growth medium and in infected mammalian cells [8],[14]. At the top of the cascade is the TetR-type regulator BspR that stimulates the expression of *bprP*. The *bspR* gene is located on chromosome 1 of the *B. pseudomallei* genome whereas *bprP* is part of the T3SS3 gene cluster on chromosome 2 [14]. The ToxR-like BprP in turn activates genes encoding the structural components of T3SS3, including the *araC*-type regulatory gene *bsaN*. BsaN is important for the activation of T3SS3 effector and translocon gene expression, and several regulatory genes including *bprC* and *virAG*, whose gene products control T6SS1 expression [8]. The mechanisms through which these transcriptional regulators control the expression of their target genes are not understood. It is also unclear whether these regulators are acting directly on the identified target genes or through as yet undiscovered intermediary regulators, and whether additional host cell cofactors are involved that may serve as intracellular signals.

Compared to T3SSs in other pathogens such as *Pseudomonas, Salmonella* and *Shigella*, only a limited number of effectors have been identified for *B. pseudomallei* T3SS3. One of the effector proteins secreted by T3SS3 is BopE, which is annotated to exhibit guanine nucleotide exchange factor activity and has been reported to facilitate invasion of epithelial cells [15]. *bopA* is generally assumed to encode a T3SS3 effector since it is located adjacent to *bopE*, although T3SS3-dependent secretion of BopA has never been verified. Functionally, BopA has been described to promote resistance to LC3-associated autophagy and a *bopA* mutation results in an intracellular replication defect [16],[17]. A third effector protein, BopC (BPSS1516), was recently shown to be secreted via T3SS3, and *bopC* mutants were reported to be less invasive in epithelial cells [18] and to exhibit delayed endosome escape and reduced intracellular growth in J774 murine macrophages [19].

To determine the full extent of the BsaN regulon and examine whether BsaN activates the expression of additional effector genes, we performed global transcriptome analysis of *B. pseudomallei* KHW wildtype (WT) and a Δ*bsaN* mutant strain using RNAseq. Our analysis shows that 111 genes are under the direct or indirect transcriptional control of BsaN. In addition to activating loci associated with T3SS3, we demonstrate that BsaN functions to repress transcription of other loci. Thus, BsaN functions as a central regulatory factor within a more extensive network to facilitate the intracellular lifecycle of *B. pseudomallei*.

## Results

### Identification of the BsaN regulon through RNAseq analysis

BsaN (*BPSS1546* in the reference *B. pseudomallei* K96243 genome) was previously shown to function as a central regulator of a hierarchical cascade that activates effector and translocon genes of T3SS3 as well as several associated regulatory genes [8],[14]. Furthermore, BsaN was shown to activate the expression of certain T6SS1-associated genes including the two-component regulatory system locus *virAG* (*BPSS1494, 1495*), and the *bim* actin motility genes (*BPSS1490-1493*). To gain further insight into the BsaN regulon, massive parallel sequencing was performed on cDNA prepared from RNA isolated from wild-type and Δ*bsaN* mutant strains. We had previously shown that complementation of our Δ*bsaN* mutant with a *bsaN* plasmid could restore the secretion of the BopE effector [14], showing that our complementation restored protein expression of the effectors and that the mutation was specific to *bsaN* and not due to off target effects.

Between 16 and 56 million reads (n = 2 from 3 combined cultures) were obtained that aligned to non-ribosomal genes in the KHW [20] genome (Additional file 1: Table S1). Reads of the technical replicates displayed high reproducibility (R-value) (Additional file 1: Table S1) demonstrating that variability was not introduced through sample preparation or sequencing errors. The K96243 reference genome was co-aligned for ease of gene annotation. The nucleotide sequences of chromosomes I and II are 99.3 and 99.1% identical, respectively.

**Figure 1:**
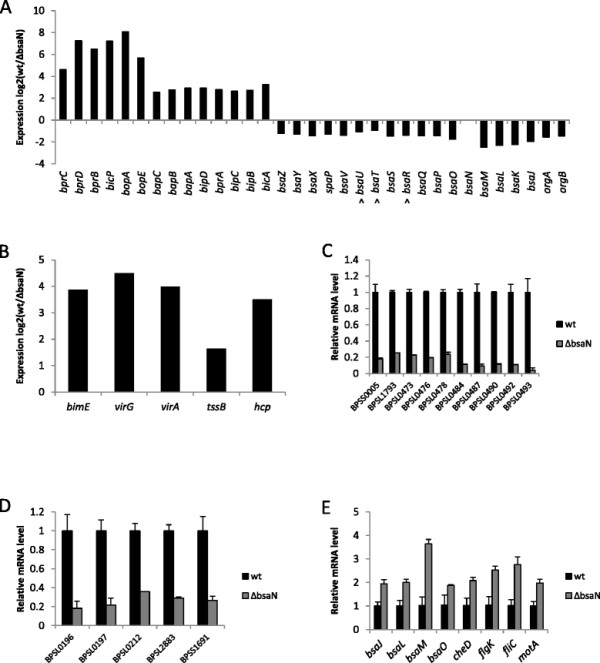
**Regulation of selected genes by BsaN as analyzed by RNAseq and qRT-PCR. A**. Activation and repression of T3SS3 cluster genes as analyzed by RNAseq. The adjusted p value for all genes is less than 0.01 with the exception of three genes denoted with ^. **B**. Activation of BsaN regulated T6SS1 and *bim* motility genes as analyzed by RNAseq. **C** and **D** qRT-PCR validation of selected activated genes. Expression of each in wild-type *B. pseudomallei* KHW gene is set to 1; transcription was normalized to that of the *recA* reference gene. **E**. qRT-PCR validation of repressed genes. Expression of each in wild-type *B. pseudomallei* KHW gene is set to 1; transcription was normalized to that of the 16S rRNA reference gene. The *flgL* gene is located upstream and in the same transcriptional unit as *flgK*.

Comparison between wild-type and Δ*bsaN* transcriptomes identified 111 genes that were differentially regulated using 3-fold or more (adjusted p-value < 0.01) as the cut off. Of these, 60 genes were expressed more highly in wild-type KHW compared to the Δ*bsaN* strain, indicating that BsaN directly or indirectly activates their transcription (Table 1). However, 51 genes were expressed more highly in the Δ*bsaN* mutant suggesting that BsaN can function directly or indirectly as a repressor (Table 2). RNAseq results were validated using quantitative real time-PCR (qRT-PCR) analysis for select loci. RNAseq analysis identified all genes that we had previously shown to be activated by BsaN [8],[14] (Figure 1A and 1B, Table 1). The effector and chaperone genes *bopE*, *bopA* and *bicP* together with the regulatory gene *bprD* were amongst the highest activated genes (50-270-fold). In addition, two putative transposase genes separating the T3SS3 genes and the T6SS1 gene clusters were highly activated by BsaN (Table 1). Genes activated at lower levels (3-4-fold) include a hybrid non-ribosomal peptide synthase (NRPS)/polyketide synthase (PKS) locus consisting of 22 genes (*BPSL0472-BPSL0493*) unique to *B. pseudomallei* and *B. mallei*. NRPS/PKS systems are found in microbes and fungi, and are generally responsible for the production of complex natural compounds such as antibiotics and siderophores. *Burkholderia* species are rich in NRPS/PKS loci that contain multiple metabolic genes or encode large multidomain synthases [21]. Although the precise function of this NRPS/PKS locus is not currently known, the presence of a diaminobutyrate-2-oxoglutarate amino transferase gene (*BPSL0476*) suggests that 2,4-diaminobutrate is one of the polyketide’s component. Loci for methionine and threonine biosynthesis, as well as ribose uptake (Table 2), were activated at similar levels. Representative BsaN-activated genes were confirmed by qRT-PCR (Figure 1C-D).

**Table 1:** **List of 60 genes that are expressed 3-fold and higher in the wild-type versus Δ** ***bsaN*** **mutant strains (p < 0.01)**

**Gene locus ID**	**Gene**	**Protein description**	**Fold activation**
T3SS3 associated			
BPSS1512	*tssM*	ubiquitin-specific proteinase	9.3
BPSS1513			7.5
BPSS1514	*folE*	GTP hydrolase	5.1
BPSS1515			9.0
BPSS1516	*bopC*	T3SS-3 effector	48.2
BPSS1518		transposase	44.3
BPSS1519		transposase	10.1
BPSS1523	*bicP*	T3SS-3 chaperone	149.0
BPSS1524	*bopA*	T3SS-3 effector	269.4
BPSS1525	*bopE*	T3SS-3 effector	51.7
BPSS1526	*bapC*	T3SS-3 effector	5.9
BPSS1527	*bapB*	T3SS-3 effector	6.8
BPSS1528	*bapA*	T3SS-3 effector	7.6
BPSS1529	*bipD*	T3SS-3 translocon	7.6
BPSS1531	*bipC*	T3SS-3 translocon	6.3
BPSS1532	*bipB*	T3SS-3 translocon	6.6
BPSS1533	*bicA*	T3SS-3 chaperone	9.4
T6SS1 apparatus			
BPSS1497	*tssB*	T6SS-1	3.1
BPSS1498	*hcp*	T6SS-1	11.3
Actin based motility			
BPSS1490		N-acetylmuramoyl-L-Ala-amidase	13.5
BPSS1491		ADP-heptose:LPS transferase	8.8
BPSS1492	*bimA*	Bim actin polymerization protein	7.8
BPSS1493			14.5
Polyketide biosynthesis			
BPSL0472-BPSL0493		NRPKS/PKS biosynthesis locus	3.0-4.3
BPSL2883		Glyoxalase/bleomycin resistance protein/dioxygenase	4.0
Amino acid biosynthesis and sugar uptake			
BPSL0196	*metW*	methionine biosynthesis protein MetW	4.2
BPSL0197	*metX*	homoserine O-acetyltransferase	3.4
BPSS1691	*metZ*	O-succinylhomoserine sulfhydrylase	3.2
BPSS0005	*kbl*	2-amino-3-ketobutyrate CoA ligase	6.3
BPSS0006	*tdh*	L-threonine dehydrogenase	5.5
BPSL1793		Periplasmic binding protein (ribose binding)	3.4
Regulatory			
BPSS1494	*virG*	T6SS-1 response regulator	22.4
BPSS1495	*virA*	T6SS-1 His kinase	15.8
BPSS1520	*bprC*	T3SS-3 AraC-type regulator	24.5
BPSS1521	*bprD*	T3SS-3 regulator	151.5
BPSS1522	*bprB*	T3SS-3 response regulator	89.5
BPSS1530	*bprA*	T3SS-3 HNS-type regulator	6.9
BPSL0480	*syrP*	NPKS/PKS regulator	3.9

**Table 2:** **List of 51 genes that are expressed 3-fold and lower in the wild-type versus Δ** ***bsaN*** **mutant strains (p < 0.01)**

**Gene locus ID**	**Gene**	**Protein description**	**Fold repression**
T3SS3 apparatus			
BPSS1545	*bsaO*		−3.3
BPSS1547	*bsaM*		−5.6
BPSS1548	*bsaL*		−5.0
BPSS1549	*bsaK*		−4.7
BPSS1550	*bsaJ*		−3.9
BPSS1551	*orgA*		−3.0
Flagella-dependent motility			
BPSL0281	*flgL*	Flagellar hook-associated protein	−3.3
BPSL3319	*fliC*	Flagellin	−3.7
BPSL3320	*fliD*	Flagellin	−3.0
BPSL3321		Unknown	−3.1
Polyketide biosynthesis			
BPSS0130		Non-ribosomal peptide synthase	−3.1
BPSS0303-BPSS0311		PKS biosynthesis locus	−3.0 - (−6.1)
BPSS0328		Malate/L-lactate dehydrogenase	−7.8
BPSS0329		Fatty aldehyde dehydrogenase	−9.6
BPSS0330		Amino acid transporter	−19.7
BPSS0331		Dihydrodipicolinate synthase	−19.0
BPSS0332		Hydroxyproline-2-epimerase	−21.7
BPSS0333		Deaminating oxidase subunit	−18.8
BPSS0334		Deaminating oxidase subunit	−24.7
BPSS0335		Deaminating oxidase subunit	−20.1
BPSS0337			−3.0
BPSS0338		Transposase	−12.0
BPSS0339		4-Hydroxyphenylpyruvate	−8.2
Lipid metabolism			
BPSS2037		Inner membrane fatty acid desaturase	−3.0
BPSS2038		Acyl carrier protein	−3.4
BPSS2039		Cyclopropane-fatty-acyl-phospholipid synthase	−3.6
BPSS2040		Inner membrane fatty acid desaturase	−3.2
Energy metabolism			
BPSL1744	*arcB*	Ornithine carbamoyltransferase	−3.4
BPSL1745	*arcC*	Carbamate kinase	−3.2
BPSL2404		Periplasmic ligand binding protein	−7.3
BPSL2405		FAD-dependent deaminase	−5.4
BPSS1885		Aromatic hydrocarbons catabolism-related reductase	−3.1
BPSS1886		Aromatic hydrocarbons catabolism-related dioxygenase	−4.2
BPSS1887		Aromatic oxygenase	−3.1
BPSS1888		Aromatic oxygenase	−3.0
BPSL2380	*cyoC*	Cytochrome *bo* oxidase subunit	−3.4
BPSL2381	*cyoD*	Cytochrome *bo* oxidase subunit	−3.0
Regulatory			
BPSS0336		AraC-type regulator, adjacent to polyketide genes	−8.1
Adaptation			
BPSL3369	*acoD*	Glycine betaine aldehyde dehydrogenase	−4.0

Intriguingly, genes encoding the T3SS3 apparatus components were found to be repressed in the wildtype compared with the Δ*bsaN* mutant, suggesting a role for BsaN in limiting apparatus synthesis when translocon and effector genes are transcribed (Figure 1A, 1E, Table 2). Also repressed are polar flagellar motility loci on chromosome 1 including the flagellin genes *fliC* and *fliD,* as well as flagellar hook proteins *flgL* and *flgK.* Repression of these genes as well as *motA* (*BPSL3309*) and *cheD* (*BPSS3302*) were validated by qRT-PCR (Figure 1E). In *Salmonella* and other bacteria, *motAB* are key components of the flagellar motor complex [22]. *motAB* in KHW are part of a chemotaxis (*che*) locus, which is repressed 2–2.9-fold (p < 0.01) as assessed by RNAseq. In addition, expression of a second polyketide biosynthesis locus (*BPSS0303-BPSS0311*) was reduced in a *ΔbsaN* mutant, possibly by repression of a co-localized *araC*-type regulatory gene, *BPSS0336* (Table 2). However, down-regulation of this cluster could not be verified by qRT-PCR (data not shown). We were likewise unable to validate repression of *BPSL2404-2405*, which putatively encode transport and energy metabolism functions, respectively, in addition to *BPSS1887-1888*, which are postulated to encode oxidative enzymes for energy metabolism.

Additional loci implicated in lipid and energy metabolism are also repressed (Table 2). Catabolic genes encode a cytochrome *o* oxidase typically used by bacteria in an oxygen-rich environment [23], along with enzymes involved in the aerobic degradation of aromatic compounds and in the degradation of arginine. A gene involved in the synthesis of betaine, an osmoprotectant which serves to adapt Gram-negative bacteria to conditions of high osmolarity, is also repressed by BsaN.

### BsaN together with chaperone BicA directly activate T3SS3 effector and T6SS1 regulatory genes

We have previously shown that expression of the two component regulatory system *virAG* and the genes from *BPSS1520* (*bprC*) to *BPSS1533* (*bicA*) in the T3SS3 cluster were regulated by BsaN in concert with the chaperone BicA [14]. To determine whether BsaN/BicA activate these genes directly, *bsaN* and *bicA* open reading frames (orfs) from *B. pseudomallei* strain KHW were inserted into a plasmid downstream of an arabinose-inducible promoter on pMLBAD [24]. These constructs were introduced into *E. coli* DH5α [25] along with an additional construct containing putative promoter regions of several BsaN target genes transcriptionally fused to *lacZ* on pRW50 [26] or pRW50mob, which contains the oriT fragment for pOT182 [27]. The effect of BsaN/BicA on promoter activity was then assessed by β-galactosidase activities.

**Figure 2:**
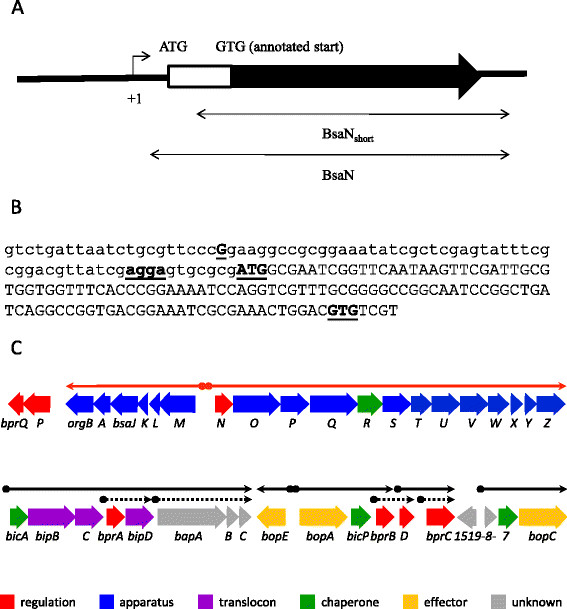
**Transcriptional analysis of*bsaN*and BsaN/BicA-regulated genes. A**. Schematic diagram of the *bsaN* gene. Arrow above +1 indicates the transcriptional start site and direction. Double-headed arrows indicated the DNA fragments used for the reconstitution of BsaN-mediated promoter activation experiments. **B**. Promoter region indicating the transcriptional start site and start codon of *bsaN*. Bold and underlined letter G indicates the transcriptional start site (+1 in 2A). Bold and underlined agga indicate the putative RBS. Bold and underlined ATG and GTG indicate the actual and wrongly annotated start codons of *bsaN*, respectively. **C**. Genetic and transcriptional organization of T3SS3 genes. Arrows indicate transcriptional units. Putative promoter regions are depicted as shaded spheres at the beginning of line arrows. Red line arrows denote operons regulated by BprP. Black line arrows indicate operons regulated by BsaN. Black dotted arrows with shaded diamonds represent putative promoters that were analyzed for direct activation by BsaN/BicA, however, no expression was found (Additional file 1: Table S2).

**Figure 3:**
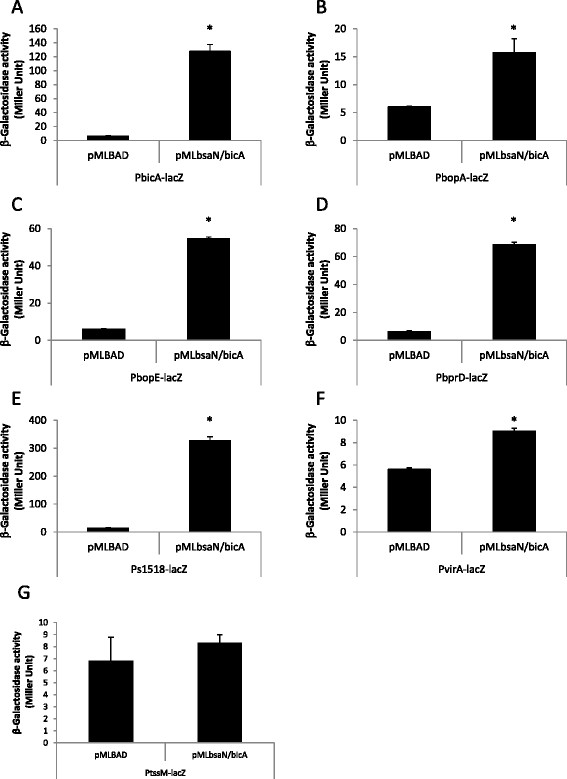
**Activation of promoters by BsaN/BicA in*E. coli.*** The ability of BsaN and BicA to directly activate the expression of promoters was examined by providing regulatory genes in *trans* and measuring β-galactosidase activities arising from the expression of transcriptional promoter*-lacZ* fusions in *E. coli* DH5α. Effect of BsaN/BicA on the expression of **A**. P*bicA-lacZ* fusion, **B**. P*bopA-lacZ* fusion, **C**. P*bopE-lacZ* fusion, **D**. P*bprD-lacZ* fusion and **E**. Ps1518*-lacZ* fusions; Ps1518 denotes the promoter region of BPSS1518. Effect of BsaN/BicA on the expression of **F**. P*virA-lacZ* fusion and **G**. P*tssM*-*lacZ* fusion. *p < 0.05.

The putative *bsaN* orf is annotated in the *B. pseudomallei* genome database to initiate from a GTG start codon [28]. We identified a second potential start codon (ATG) and ribosome binding site 117 nucleotides (nt) upstream of GTG (Figure 2A, B). *bsaN/bicA* expression constructs (Figure 2A) that were initiated from GTG were unable to activate transcription of *bicA, bopA* and *bopE* in *E. coli* (Additional file 1: Table S2), supporting the notion that the ATG was the actual start codon for BsaN. Furthermore, a transcriptional start site was identified 56 nucleotide upstream of the ATG codon via RNA ligase-mediated rapid amplification of cDNA ends (RLM-RACE) (Figure 2B). A putative Ribosomal Binding Site (RBS) is located in front of the ATG condon. Replacing the GTG-initiated *bsaN* orf with the longer version containing the ATG start site resulted in activation of the *bicA, bopA* and *bopE* promoters as well as those for *BPSS1521* (*bprD*)*, BPSS 1495* (*virA*) and the putative transposase *BPSS1518* (Figure 3A-F). Expression of BsaN alone was not sufficient to activate these promoters (Additional file 1: Table S2), demonstrating the co-requirement for BicA. No apparent BsaN/BicA-dependent promoter activity was obtained for *BPSS1528* (*bapA*)*, BPSS1523* (*bicP*)*, BPSS1530* (*bprA*)*,* or *BPSS1520 (bprC)* (Additional file 1: Table S2) (refer to Figure 2C for gene location). Furthermore, BsaN/BicA could not activate transcription of a *BPSS1512* (*tssM)-lacZ* fusion in *E. coli* (Figure 3G). Thus, BsaN/BicA drives the expression of *bprDC* and the *BPSS1518-1516* operons directly, whereas *bicP* and *bprB* gene expression is likely driven by the upstream-located *bopA* promoter. Transcription of the *bapABC* and *bprA* genes could be driven from the *bicA* promoter. Collectively, these results are represented in Figure 2C where the five validated promoters and operon structures controlled directly by BsaN/BicA are depicted by black solid line arrows.

### Identification of transcriptional start sites and the sequence motif for BsaN/BicA activation

**Figure 4:**
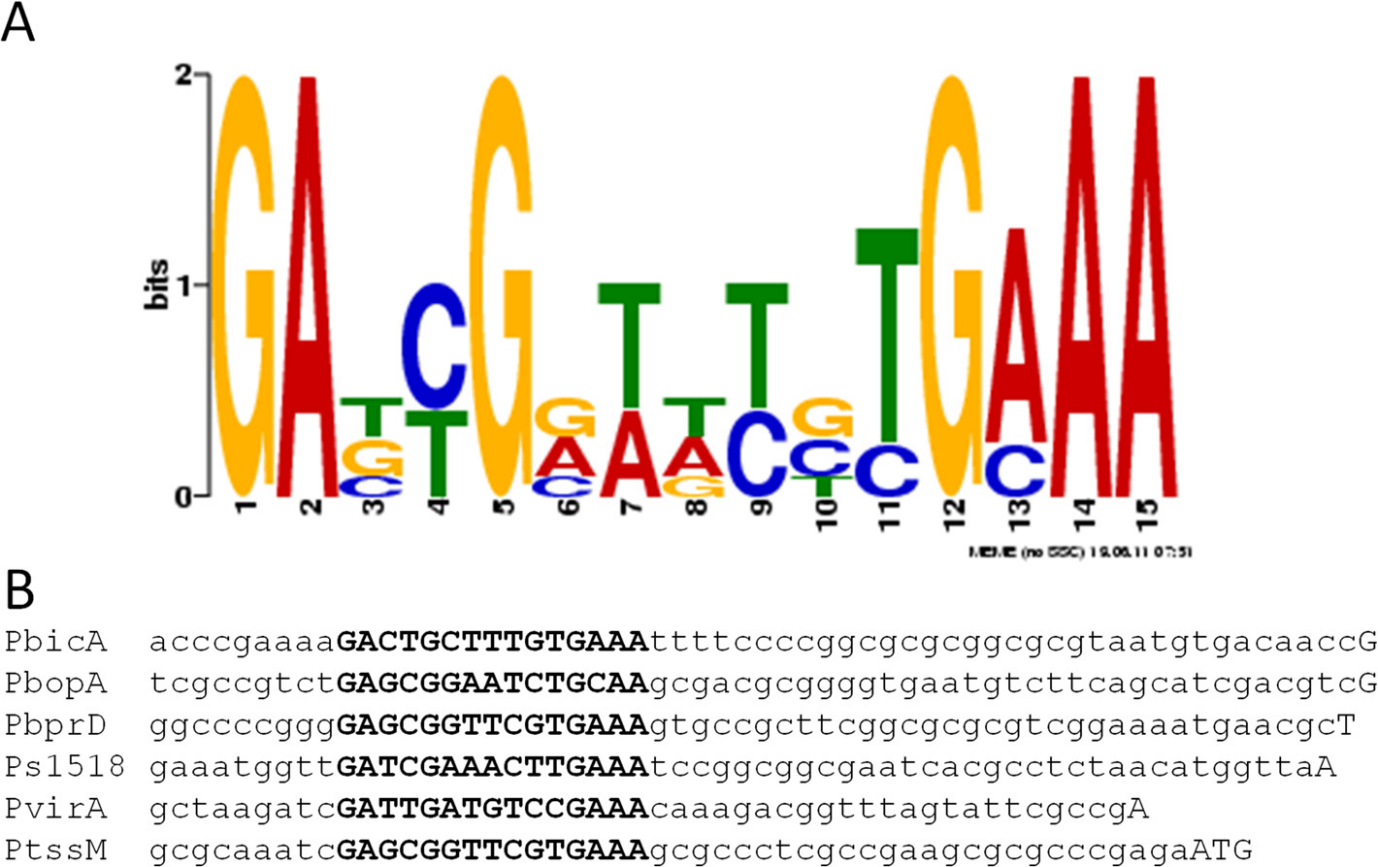
**Sequence motifs in promoter regions of BsaN/BicA-regulated genes. A**. The sequence motif for the BsaN box as indicated in bold, capital letters was identified using the bioinformatics tool MEME. **B**. The sequence of the BsaN box generated by MEME from the 5 BsaN-activating promoters as denoted in capital letters. The 3’capitalized letters denote the start of transcription with the exception of P*tssM,* which is the translational start codon of TssM.

Similarities between BsaN/BicA regulated promoters were examined by first determining their transcriptional start sites using RLM-RACE. One transcriptional start site was identified for the *bicA, bprD* and *BPSS1518* promoters, and two start sites were detected for the *bopA* and *virA* promoters. We were unable to identify a transcriptional start site for *bopE*, which is divergently transcribed from *bopA* (Figure 2C). A 150-bp sequence upstream of each transcriptional start site was submitted to MEME (Motif Elicitation for Prediction of DNA Motifs), which identified a 15 bp motif that we designated as the putative BsaN box (Figure 4A). The distance from the transcriptional start site varied from 24 bp (*virA*) to 35 bp (*bicA* and *bopA*) (Figure 4B). When the motif was submitted to Motif Alignment & Search Tool (MAST) to search for other potential BsaN/BicA-regulated promoters in the *B. pseudomallei* genome (strain K96243), BsaN boxes were also found upstream of *tssM* and *BPSS1889*, a putative gene encoding an AraC family protein, in addition to those already identified. However, qRT- PCR analysis of *BPSS1889* expression in Δ*bsaN* and Δ*bicA* mutants did not reveal a decrease in expression compared to wild-type bacteria (data not shown). *BPSS1889* is located adjacent but transcribed in the opposite direction to the operon *BPSS1884-1888,* which was shown by RNAseq to be repressed by BsaN (Table 2). Although we could not confirm BsaN-dependent regulation of *BPSS1889* by qRT-PCR, the upstream BsaN box suggests the possible involvement of this putative regulator in repression of the operon *in vivo*. It is likely that conditions for BsaN-dependent repression are difficult to establish *in vitro* resulting in variability and lack of validation. We also could not identify any −10 and −35 sequences for prokaryotic housekeeping sigma factor in these promoters. It is likely that the BsaN/BicA-regulated promoters are transcribed by one or more alternative sigma factors. Unfortunately, *B. pseudomallei* genome harbours more than 10 alternative sigma factors that have not been systematically studied. Therefore, their recognition sequences are currently unknown.

*tssM* is one of the highly activated genes in our RNAseq analysis (Table 1) confirming previous *in vivo* expression studies [29]. However, despite the presence of the BsaN box upstream of the putative *tssM* operon (*BPSS1512-1514*), BsaN/BicA alone is not sufficient to activate *tssM* transcription in *E. coli* (Figure 3G). This suggests that *tssM* regulation is more complex and likely requires additional cis and/or trans-acting regulatory elements for activation.

### Determining the sequence motif requirement for BsaN/BicA activation

**Figure 5:**
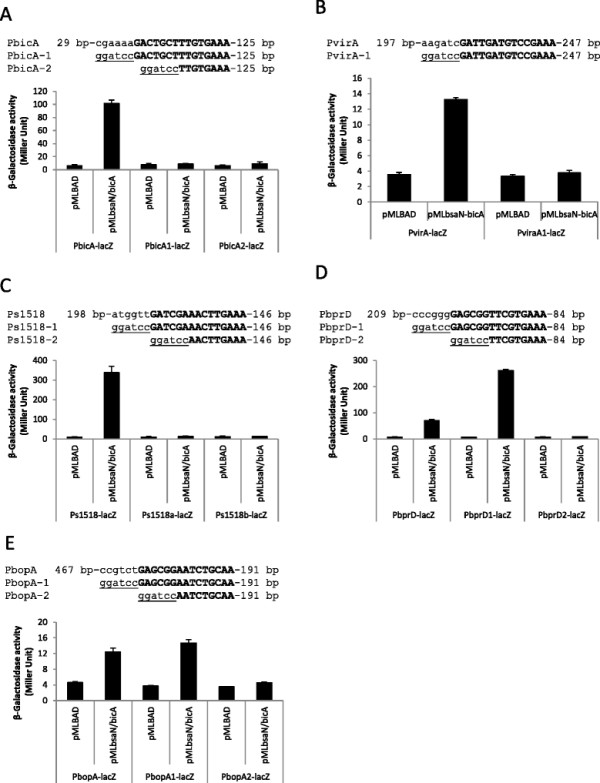
**Analysis of BsaN box requirements for transcription activation by BsaN/BicA.** The ability of BsaN/BicA to directly activate the expression of truncated promoters was examined by providing regulatory genes in *trans* and measuring β-galactosidase activities arising from the expression of transcriptional promoter*-lacZ* fusions in *E. coli* DH5α. The top sequence of each gene includes the intact promoter region; sequence 1 is deleted up to the BsaN box; sequence 2 also includes a 6 nucleotide deletion of the BsaN box. Effect of BsaN/BicA on the expression of **A**. P*bicA-lacZ* fusion, **B**. P*virA-lacZ* fusion and **C**. Ps1518*-lacZ* fusion; Ps1518 denotes the promoter region of BPSS1518. Effect of BsaN/BicA on the expression of **D**. P*bprD-lacZ* fusion and **E**. P*bopA-lacZ* fusion.

To determine whether the putative BsaN box motif was required and sufficient for the other genes regulated by BsaN/BicA, we constructed two types of truncated promoter-*lacZ* fusions. The “type 1” deletion contained only the BsaN motif and lacked all upstream sequences. The “type 2” deletion lacked all upstream sequences in addition to the first six bp of the putative BsaN box motif. We assayed the ability of these truncated promoters to drive *lacZ* expression in the presence of BsaN/BicA. All truncated versions of the promoter regions for *bicA*, *virA* and *BPSS1518* lost promoter activity (Figure 5A-C). In contrast, versions containing the intact BsaN box for *bprD* (Figure 5D) and *bopA* (Figure 5E) were still functional, but further truncation eliminated their activation. The type 1 truncated version of the *bprD* promoter (P*bprD1-lacZ*) had three-fold higher β-galactosidase activity than the full length promoter- *lacZ* fusion (P*bprD-lacZ)* (Figure 5D)*,* whereas deletion of sequences upstream of the *bopA* promoter did not have a significant effect on the level of activation (Figure 5E).

### BprP directly activates *bsaN* and *bsaM*

**Figure 6:**
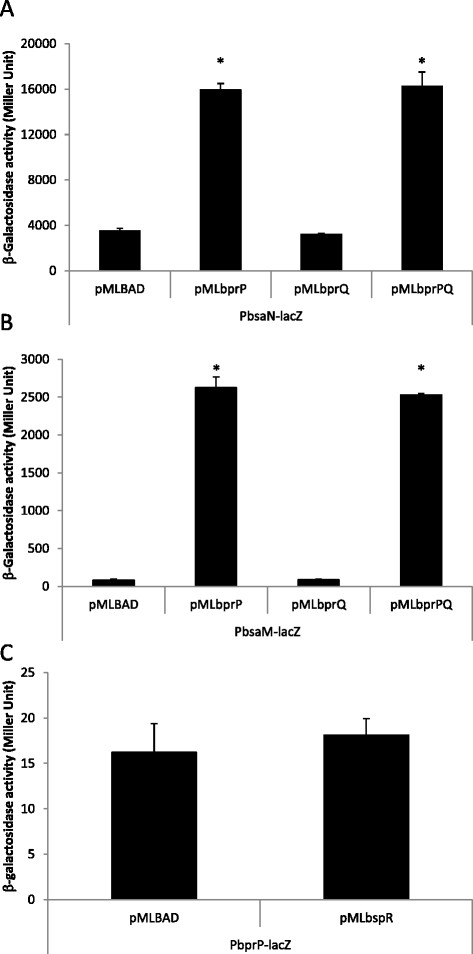
**Activation of*bsaM*and*bsaN*promoters by BprP in*E. coli.*** The ability of BprP to directly activate the expression of promoters in the presence and absence of BprQ was examined by providing the *bprP* and *bprQ* genes in *trans* and measuring β-galactosidase activities arising from the expression of transcriptional promoter*-lacZ* fusions in *E. coli* DH5α. **A**. Effect of BprP and BprQ on the expression of P*bsaN-lacZ* fusion. **B**. Effect of BprP and BprQ on the expression of P*bsaM-lacZ* fusion. **C**. Effect of BspR on the expression of P*bprP-lacZ* fusion. *p < 0.05.

In the hierarchical control of T3SS3 and T6SS1 expression, BspR was suggested to activate the expression of *bprP*[14]. Previously, BprP was shown to bind sequences upstream of *bsaM* and *bsaN* (refer to Figure 2C for gene location) [14], suggesting that it directly activates their transcription. *bsaN* is the first orf of the putative operon that encodes structural components of T3SS3 (Figure 2C) and is divergently transcribed from *bsaM*. To better understand how *bsaN* expression itself is controlled, we examined the relationships to its upstream regulators BspR and BprP using the LacZ fusion assay as described previously [8]. Plasmids with either *bspR* or *bprP* under arabinose induction control were introduced into *E. coli* containing plasmids with either a *bsaN*-*lacZ* fusion or a *bsaM*-*lacZ* fusion. A *bprP-lacZ* fusion served as control for BspR regulation. The ability of BspR and BprP to directly activate *bsaN-lacZ, bsaM-lacZ* and *bprP-lacZ* expression was determined by measuring β-galactosidase activity. As expected, BprP activated both the *bsaM* and *bsaN* promoters in *E. coli* (Figure 6A, B). The presence of *bprQ*, a gene immediate downstream from *bprP*, had no effect on the activity of BprP. Furthermore, BprP did not activate its own promoter in *E. coli* (data not shown). However, BspR was not able to activate the promoter of *bprP* demonstrating that this regulator is not active in *E. coli* or that additional factors are required for activation (Figure 6C).

### Analysis of BsaN/BicA-regulated virulence loci

BsaN/BicA directly induces the expression of three known T3SS3 effector loci, *bopA*, *bopC* and *bopE*. Recent studies suggest that the T3SS3 effectors BopC and BopE are involved in invasion of epithelial cells and endosome escape [15],[18],[19], while BopA has been implicated in escape from autophagy [17]. BopC was recently shown to be secreted via T3SS3 in *B. pseudomallei* K96243 [18], and our data confirm this (Additional file 1: Figure S1). In *B. pseudomallei* KHW, mutation of *bopA*, *bopC* or *bopE*[30] individually resulted in no detectable difference in numbers of bacteria inside RAW264.7 mouse macrophages when measured 2 hr. after infection (Additional file 1: Figure S2A). Upon extended incubation times, however, the Δ*bopA* and the Δ*bopACE*[30] strains exhibited an intracellular replication defect that was intermediate between levels observed for wildtype KHW and the Δ*bsaM*[30] or Δ*bsaN* mutant derivatives. No differences in intracellular growth or host cell cytotoxicity were observed for the *bopC* or *bopE* mutant strains, although infection with the *bopA* or *bopACE* triple deletion mutants resulted in a decrease in cytotoxicity (Additional file 1: Figure S2B) that coincided with a reduction in the rate of intracellular replication (Additional file 1: Figure S2A), suggesting that intracellular replication results in host cell toxicity. This is in contrast to the T3SS3 Δ*bsaM* and the Δ*bsaN* regulatory mutants in strain KHW, which are limited in their ability to multiply intracellularly as previously reported (Additional file 1: Figure S2A).

Three BsaN/BicA-activated orfs are located between the T3SS3 and T6SS1 loci, and upstream of the T3SS3 effector gene *bopC.* We analyzed these orfs for potential roles in intracellular replication and cell-to-cell spread. *BPSS1512* encodes TssM, was previously shown to be secreted independently of T3SS3 and T6SS1 and functions as a broad-base deubiquitinase, with activity on TNFR-associated factor-3, TNFR-associated factor-6, and IκBα [31]. *BPSS1513* is predicted to encode a short (97 aa) protein of unknown function and was not secreted under our assay conditions (Additional file 1: Figure S3A). *folE (BPSS1514)* encodes a putative GTP cyclohydrolase I, suggesting a role in tetrahydrofolate biosynthesis rather than in virulence. Consistent with this notion, Δ(*BPSS1513*-*folE)* mutant did not exhibit defects in cell-based virulence assays (Additional file 1: Figure S3B-E).

## Discussion

T3SSs and T6SSs play important roles in bacterial-host cell interactions [32],[33]. As each system is a complex structure encoded by 20 or more genes, it is expected that their expression and assembly would be tightly regulated. In *B. pseudomallei*, T3SS3 and T6SS1 gene clusters are highly induced following host cell infection [8], and their function is critical for virulence in animal models [8],[13]. T3SS3 has been shown to promote escape from endocytic vesicles, and T6SS1 plays a key role in promoting intercellular spread by fusion of adjacent cell membranes, leading to the formation of MNGCs that can be found in melioidosis patients [34]. Upregulation of T3SS3 is mediated by a signalling cascade initiating from BspR through BprP, which in turn increases the expression of the AraC-type regulator BsaN. Using global transcriptome and promoter activation analysis, we have shown that the BsaN regulon occupies a central position in modulating the expression of T3SS3, T6SS1 and several additional loci that are likely involved in promoting virulence and intracellular survival.

Regulatory factors may act to control expression by acting directly on a given gene, or indirectly by modulating a regulatory intermediate. We found that BsaN in complex with the T3SS3 chaperone BicA directly controls the expression of 19 loci in a region of chromosome 2 containing T6SS1 and T3SS3 accessory genes (*BPSS1494-BPSS1533*). BsaN/BicA activated transcription of the operons encoding T3SS3 effector proteins, the BipBCD translocon complex, chaperones, and other transcriptional regulators, as well as two genes of unknown function (*BPSS1513-1514*). BsaN/BicA upregulates expression of T6SS1 by activating the transcription of the two component regulatory system loci *virAG* and *bprC,* which in turn induce the *hcp* and *tssAB* loci, encoding T6SS1 tube and sheath proteins [8],[35]. Interestingly, our RNAseq and qRT-PCR analyses revealed that BsaN also acts to repress transcription of T3SS3 apparatus genes in the *bsaM* and *bsaN* operons that are otherwise directly activated by the upstream regulator BprP. It is possible that BsaN mediates repression indirectly as the *bsaM* and *bsaN* intergenic region lacks a recognizable BsaN binding motif (see below). It is unlikely, however, that repression occurs due to decreased expression of *bprP* since its transcription is unchanged in a Δ*bsaN* mutant. Taken together, these findings demonstrate that BsaN plays a dual role in the regulation of T3SS3; one in coordinating translocon and effector transcription, and a second in preventing costly synthesis of T3SS3 apparatus components that are no longer required. Given the critical role of T3SS3 and T6SS1 in causing disease, BsaN/BicA could be considered a central regulator of *B. pseudomallei* mammalian virulence. Virulence studies in mice support this notion, since the Δ*bsaN* mutant was unable to cause disease [8] in contrast to the Δ*bspR* mutant, which produced a more chronic infection in mice compared to wildtype bacteria [14].

In addition to loci associated with T3SS3 and T6SS1, 41 other genes with potential roles in virulence were also found by RNAseq to be positively regulated by BsaN, most notably the *bimBCAD* intracellular motility operon and *tssM*. Regulation of *bimA* has been shown to be through *virAG*[8], explaining why no BsaN motif was identified for the operon. While *bimA* encodes an autotransporter protein that nucleates and polymerizes host cell actin to facilitate intracellular motility and cell-cell spread by the bacteria [36], the functions of the other loci in the *bim* operon are unknown. TssM has been shown to suppress host NFκB and Type I interferon pathways [31]. TssM is expressed and secreted inside cells following infection with *B. mallei*[29], however, secretion occurs independently of T3SS3 and T6SS1 [31]. BsaN was also found to activate expression of a putative non-ribosomal peptide synthase (NRPS)/polyketide synthase (PKS) biosynthesis locus. The diversity of polyketides, PKSs and NRPS/PKS hybrid systems was recently reviewed by Hertweck [37]. The *B. pseudomallei* locus is similar in gene content to that of a recently described plasmid encoded NRPS/PKS system in the marine bacterium *Alteromonas macleodii*, which was suggested to produce a bleomycin-related antibiotic Unlike *A. macleodii*, the gene encoding the putative bleomycin-family resistance protein (*BPSL2883*) is not co-localized with the NRPS/PKS gene cluster, although they are similarly regulated by BsaN (Table 1).

BsaN is homologous to the *Salmonella typhimurium* InvF, *Shigella flexneri* MxiE and the *Yersinia enterocholitica* YsaB transcriptional regulators [38]–[40]. All belong to the AraC/XylS family of transcriptional regulators, which act in complex with a chaperone to activate their respective T3SS genes. The chaperones not only serve as cognate partners to the transcriptional activators but also pair with T3SS translocase proteins, which are secreted into the host membrane to facilitate the injection of effector proteins [41]. We currently, have no understanding of the timed mechanism that frees BicA and allows it to partner with BsaN. The *S. typhimurium* chaperone SicA was shown to partition the translocase SipB and SipC, and it is sequestered by SipB [42]. Once apparatus assembly is complete, translocases are secreted and SicA is free to complex and thus activate InvF. The InvF-SicA split feedback regulatory loop, which includes positive autoregulation of *invF*, is conserved in *Y. enterocholitica*[40]. However, in *S. flexneri* MxiE-dependent activity is inhibited via sequestration by the T3SS substrate OspD1 when the apparatus is inactive [43]. Only when OspD1 is secreted, can MxiE partner with its chaperone IpgC to activated transcription of effector genes.

**Figure 7:**
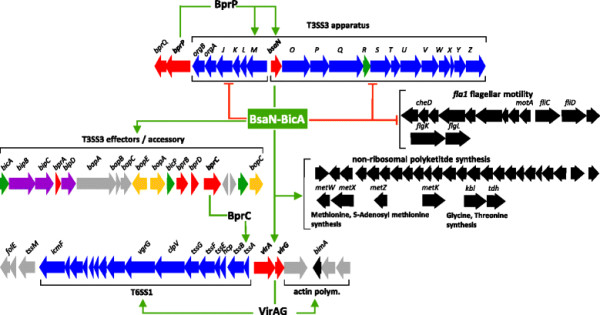
**Diagram of BsaN regulon.** The BsaN regulon is shown as part of a regulatory network, which is superseded by BprP activating transcriptions of T3SS3 apparatus genes (blue) including *bsaN*. The *bicA* gene is likely initially transcribed via read through of apparatus genes. BsaN-BicA function as a complex to activate T3SS3 translocon (purple), effector (yellow), accessory (grey) and regulatory (red) genes. Transcriptional activation is indicated by green arrows. BsaN-BicA also activate *virAG*, which in turn activates the *bimA* motility genes and the T6SS1 locus. BprC activates the T6SS1 *tssAB* apparatus genes. BsaN-BicA also activate a non-ribosomal polyketide synthesis locus and several metabolic genes. BsaN-repressed genes as indicated by red, blunted lines include T3SS3 apparatus genes and flagellar motility genes. Only genes which have been validated by qRT-PCR are shown.

Regulation by BsaN-BicA is distinct from the previously described systems. The designation of BsaN-BicA as a dual-function regulatory protein complex is illustrated by its role in activating T3SS effector and accessory genes while repressing the system’s structural and secretion components as summarized in Figure 7. BsaN was also found to suppress the transcription of 51 additional genes in the *B. pseudomallei* genome including those belonging to the *fla1* flagellar and chemotaxis locus on chromosome 1 (Figure 1E). Fla1 is the sole flagellar system in Southeast Asian *B. pseudomallei* strains such as KHW, in contrast to Australian *B. pseudomallei* isolates which possess a complete second system encoded on chromosome 2 (Fla2) [9],[44]. The conserved *fla1* locus encodes polar flagella and was shown to be responsible for swimming in liquid medium and swarming in soft agar, but played no role in intracellular motility following infection [9]. Moreover, we were intrigued to find that BsaN suppresses a second PKS/NRPS cluster (*BPSS0130, BPSS0303-BPSS0311, BPSS0328-BPSS0339*) (Table 2), where almost identical homologs were identified in *B. mallei* and *B. thailandensis* by Biggins *et al.* and shown to produce an iron-chelating siderophore called malleilactone [45]. Disruption of the MAL cluster in *B. thailandensis* reduced lethality following infection of *C. elegans*, and purified malleilactone was toxic to mammalian cells at micromolar concentrations. How the function of MAL fits within an overall regulatory framework that promotes virulence is not clear, although it is conceivable that BsaN-mediated suppression of MAL reduces the production of toxic products during infection, thereby promoting long term survival within eukaryotic hosts. Alternatively, malleilactone itself may regulate virulence factor production similar to that reported for the *P. aeruginosa* siderophore pyoverdine [46].

Until recently, BopA and BopE were the only two known T3SS effector proteins in *B. pseudomallei*. The dearth of effectors is surprising when compared to other intracellular pathogens such as *Shigella* and *Salmonella* that are known to possess numerous effectors. We have independently identified BopC (BPSS1516) as a new T3SS3 effector based on its regulatory control by BsaN/BicA. *bopC* is transcribed in an operon encoding its chaperone (*BPSS1517*) and a transposase (*BPSS1518*) that are also activated by BsaN/BicA. Incidentally, we had previously predicted by a genome-wide screen that *BPSS1516* would encode a T3SS effector based on genomic colocalization with T3SS chaperones [47]. The BsaN regulatory motif we found in the promoters of the effectors was also recently reported to be associated with T3SS3 in a condition-dependent transcriptome study [48]. Of the T3SS3-linked effector proteins; BopA, BopC and BopE, our results suggest that BopA is the most critical for promoting cellular infection, consistent with prior studies linking BopA to intracellular survival of *B. pseudomallei* and *B. mallei*[16],[17],[49]. No cellular phenotype was evident following infection with Δ*bopC* or Δ*bopE* deletion mutants, and the *ΔbopACE* triple effector mutant was indistinguishable from the Δ*bopA* single deletion strain. As with *bopE* and *bopC*, no roles were observed for the BsaN-regulated effector candidate loci *BPSS1513-1514* in cell-based virulence assays. *BPSS1513* encodes a hypothetical protein and *BPSS1514* is annotated as *folE*, a predicted GTP cyclohydrolase. Based on their genomic organization, the transcription of these loci is likely driven from the promoter upstream of *BPSS1512 tssM*. The secretion of HA-tagged BPSS1513 was not detected in *in vitro* secretion assays, although it is possible that the epitope tag could have interfered with secretion of BPSS1513, or that the assay was not performed at conditions permissive for secretion. It is intriguing why these three genes are placed under BsaN/BicA regulation by the bacterium. One possibility could be that they are important under specific stress conditions or during chronic infection.

## Conclusions

Elucidating the scope of the BsaN regulon significantly enhances our understanding of *B. pseudomallei* pathogenic mechanisms. BsaN orchestrates the temporal and spatial expression of virulence determinants during progression through the intracellular lifecycle, promoting endosome escape and possibly evasion of autophagy through activation of T3SS3 effector loci, facilitating cell-cell spread by activation of T6SS1 and the *bim* intracellular motility loci, and suppressing cellular immunity via the action of the TssM ubiquitin hydrolase. BsaN also suppresses other loci that are potentially counterproductive following intracellular localization, such as the *fla1* flagellar motility and chemotaxis locus, which could lead to activation of cellular immunity pathways through PAMP recognition. It is likely that the BsaN regulon and other virulence determinants that promote pathogenesis in higher mammals have been shaped primarily as a result of interactions with free-living protozoa, similar to what is believed to be the case for *L. pneumophila*[50]. Indeed, many of the same BsaN-regulated systems, namely T3SS and T6SS, are thought to act as “anti-predation determinants” that facilitate endosome escape and promote survival within bacteriovorus amoebae by manipulating eukaryotic pathways that are conserved from protists to humans [3]. The dual regulatory roles of BsaN – that of an activator and a suppressor – indicate that it is a key node in a regulatory program that successfully enables an environmental saprophyte to transition from the soil to surviving intracellularly.

## Methods

### Bacterial strains and culture conditions

Bacterial strains are listed in Table 3. Plasmids are listed in Table 4 and Additional file 1: Table S2. The *B. pseudomallei* wild-type strains used in this study are clinical isolates KHW. Plasmids were introduced into *E. coli* DH5α and S17-1 [51] strains by electro- or chemical-transformation. Plasmids were transferred into *B. pseudomallei* by conjugation from *E. coli* S17-1 on membrane filters. *E. coli* donors and *B. pseudomallei* recipients were first mixed on filters and incubated at 37°C on non-selective Luria-Bertani (LB) agar for 3 hours before transferring the filters onto selective media. In our RNA isolation for transcriptome analysis and qRT-PCR, *B. pseudomallei* wild-type and mutant strains were cultured in acidic (pH 5.0) RPMI medium containing 10% fetal bovine serum at 37°C for 4 hours, when bacteria were in their mid exponential growth phase (OD600 ~ 1.0). Acidification results in higher T3SS3 expression without affecting cell growth.

**Table 3:** List of strains used in this study

**Strain**	**Relevant characteristic(s)**^***a***^	**Source or reference**
***E. coli***		
DH5α	*lacZ*^−^	[25]
S17-1	Donor strain for conjugation	[51]
***B. pseudomallei***		
KHW	Clinical strain	[20]
Δ*bsaN*	KHW, *bsaN* orf was deleted	[14]
Δ*bsaM*	KHW, *bsaM* orf was deleted	[30]
Δ*bopA*	KHW, *bopA* orf was replaced by zeocin resistance gene, Zeo^r^	[30]
Δ*bopE*	KHW, *bopE* orf was deleted	[30]
Δ*bopC*	KHW, *bopC* orf was replaced by zeocin resistance gene, Zeo^r^	[30]
Δ*bopACE*	KHW, *bopA* and *bopE* orfs were deleted, *bopC* orf was replaced by zeocin resistance gene, Zeo^r^	[30]
Δ*BPSS1513-folE*	KHW, *BPSS1513* and *folE* orfs were deleted	This study

^*a*^Abbreviations: *Zeo*^*r*^ zeocin resistant.

**Table 4:** List of plasmids used in this study

**Plasmid**	**Relevant characteristic(s)**^***a***^	**Source or reference**
pMLBAD	Broad host range vector containing inducible promoter P_bad_, Tm^r^	[24]
pRW50	Low copy-number vector containing a promoter-less *lacZ* gene, Tc^r^	[26]
pOT182	Source of oriT sequence, Tc^r^	[27]
pK18mobsac	Conjugative, suicide vector containing sacB gene, Km^r^	[52]
pRW50mob	pRW50 containing oriT sequence from pOT182, Tc^r^	This study
pMLbspR	pMLBAD containing *bspR* orf from KHW, Tm^r^	This study
pMLbprP	pMLBAD containing *bprP* orf from KHW, Tm^r^	This study
pMLbprQ	pMLBAD containing *bprQ* orf from KHW, Tm^r^	This study
pMLbprPQ	pMLBAD containing *bprP* and *bprQ* orfs from KHW, Tm^r^	This study
pMLbsaNs/bicA	pMLBAD containing shorter *bsaN* orf (GTG start) and *bicA* orf from KHW, Tm^r^	This study
pMLbsaN	pMLBAD containing longer *bsaN* orf (ATG start) from KHW, Tm^r^	This study
pMLbsaN/bicA	pMLBAD containing longer *bsaN* orf (ATG start) and *bicA* orf from KHW, Tm^r^	This study
pRWbsaM	PbsaM*-lacZ* transcriptional fusion, pRW50mob containing *bsaM* upstream sequence from KHW, Tc^r^	This study
pRWbsaN	PbsaN*-lacZ* transcriptional fusion, pRW50mob containing *bsaN* upstream sequence from KHW, Tc^r^	This study
pRWbicA	PbicA*-lacZ* transcriptional fusion, pRW50mob containing *bicA* upstream sequence from KHW, Tc^r^	This study
pRWbicA1	PbicA1*-lacZ* transcriptional fusion, pRW50mob containing truncated *bicA* upstream sequence from KHW, type 1 deletion, Tc^r^	This study
pRWbicA2	PbicA2*-lacZ* transcriptional fusion, pRW50mob containing truncated *bicA* upstream sequence from KHW, type 2 deletion, Tc^r^	This study
pRWbopE	PbopE*-lacZ* transcriptional fusion, pRW50mob containing *bopE* upstream sequence from KHW, Tc^r^	This study
pRWbopA	PbopA*-lacZ* transcriptional fusion, pRW50mob containing *bopA* upstream sequence from KHW, Tc^r^	This study
pRWbopA1	PbopA1*-lacZ* transcriptional fusion, pRW50mob containing truncated *bopA* upstream sequence from KHW, type 1 deletion, Tc^r^	This study
pRWbopA2	PbopA2*-lacZ* transcriptional fusion, pRW50mob containing truncated *bopA* upstream sequence from KHW, type 2 deletion, Tc^r^	This study
pRWbprB	PbprC*-lacZ* transcriptional fusion, pRW50mob containing *bprB* upstream sequence from KHW, Tc^r^	This study
pRWbprD	PbprD*-lacZ* transcriptional fusion, pRW50mob containing *bprD* upstream sequence from KHW, Tc^r^	This study
pRWbprD1	PbprD1*-lacZ* transcriptional fusion, pRW50mob containing truncated *bprD* upstream sequence from KHW, type 1 deletion, Tc^r^	This study
pRWbprD2	PbicA2*-lacZ* transcriptional fusion, pRW50mob containing truncated *bicA* upstream sequence from KHW, type 2 deletion, Tc^r^	This study
pRWbprC	PbprC*-lacZ* transcriptional fusion, pRW50mob containing *bprC* upstream sequence from KHW, Tc^r^	This study
pRWs1518	Ps1518*-lacZ* transcriptional fusion, pRW50 containing BPSS1518 upstream sequence from KHW, Tc^r^	This study
pRWs1518a	Ps1518a*-lacZ* transcriptional fusion, pRW50 containing truncated BPSS1518 upstream sequence from KHW, type 1 deletion, Tc^r^	This study
pRWs1518b	Ps1518b*-lacZ* transcriptional fusion, pRW50 containing truncated BPSS1518 upstream sequence from KHW, type 2 deletion, Tc^r^	This study
pRWvirA	PvirA*-lacZ* transcriptional fusion, pRW50 containing *virA* upstream sequence from KHW, Tc^r^	This study
pRWvirA1	PvirA1*-lacZ* transcriptional fusion, pRW50 containing truncated *virA* upstream sequence from KHW, type 1 deletion, Tc^r^	This study
pRWtssM	PtssM-*lacZ* transcriptional fusion, pRW50 containing *tssM* upstream sequence from KHW, Tc^r^	This study

^*a*^Abbreviations: *Ap*^*r*^ ampicillin resistant, *Km*^*r*^ kanamycin resistant, *Tc*^*r*^ tetracycline resistant, *Tm*^*r*^ trimethoprim resistant.

### Bacterial mutant construction

*B. pseudomallei* gene deletions were generated by allelic exchange. Approximately 1 kb fragments upstream and downstream of the target gene were amplified from genomic DNA and cloned into pK18mobsacB vector [52] simultaneously using In-Fusion PCR cloning kit (Clontech). The plasmids were introduced into *B. pseudomallei* strains by conjugation. Homologous recombination was then selected for by growing bacteria in LB + 15% sucrose to counter select the *sacB* gene in the pK18mobsacB plasmid backbone. Successful double cross-over clones were screened by colony PCR from kanamycin sensitive colonies.

### Activation of potential promoters by regulators

The ability of regulators to directly activate the expression of promoters was examined in *E. coli* DH5α as described previously [8]. Briefly, upstream regions of *B. pseudomallei* genes encompassing at least 100 bp of non-coding sequence upstream of the start codon were amplified from KHW genomic DNA and fused to the *lacZ* gene in pRW50 or pRW50mob to generate transcriptional fusions (Table 4). Coding sequences of regulators were amplified from KHW genomic DNA and cloned into the arabinose-inducible expression vector pMLBAD. The *lacZ* fusion plasmid and arabinose-inducible regulator plasmid were introduced into the *E. coli* DH5α. β-galactosidase activities arising from the expression of promoter*-lacZ* fusions were assessed. β-Galactosidase assays were performed and values were calculated as previously described [53].

### Transcriptome analysis by RNAseq

Total RNA was extracted from three independently grown bacterial cultures that were combined at equal cell density in their exponential growth phase and quick frozen in dry ice-ethanol slurry. Approximately 2 × 10^9^ ice cold cells were centrifuged at 3000 × g for 45 sec and 4°C and RNA was isolated from cell pellets using the RiboPure™-Bacteria Kit (Ambion). Stable RNAs were removed from 10 μg RNA using the MICROBExpress kit from Ambion. Absence of genomic DNA contamination was confirmed by PCR. Paired-end libraries for Illumina sequencing [54] were prepared using the TruSeq RNA sample preparation kit version 2.0 (Illumina) according to manufacturer's High Sample (HS) protocol albeit omitting the initial poly A selection step. Libraries were generated from 2 technical replicates using 350–500 ng enriched RNA from wildtype and Δ*bsaN* mutant strains as the starting material. Library preparation and sequencing was done by the UCLA Neuroscience Genomics Core (UNGC). Reads were aligned to chromosomes I and II of *B. pseudomallei* KHW (also called BP22) (RefSeq identification numbers NZ_CM001156.1 and NZ_CM001157.1) and *B. pseudomallei* K96243 (RefSeq identification numbers NC_006350.1 and NC_006351.1) as the annotated reference genome. The number of reads aligning to each genomic position on each strand was calculated and normalized using RPKM ([reads/kb of gene]/[million reads aligning to genome]). Differentially expressed genes identified by the log2 ratio of the differential between the wildtype and Δ*bsaN* RPKMs. Only, genes with a Δlog2 value of >1.5 and < −1.5 corresponding to 3-fold up or down regulated genes with an adjusted p value (padj) of <0.01 were considered for this study.

### Measurement of *B. pseudomallei* gene expression by qRT- PCR

Expression of activated genes was confirmed by qRT-PCR of RNA prepared from bacteria grown in acidified RPMI. Gene repression was difficult to observe under these conditions; RNA for qRT-PCR analysis was therefore prepared from infected RAW264.7 cells using the following procedure: RAW264.7 cells (5 × 10^5^ cells/well) were seeded and grown overnight in DMEM medium in 12 well plates. RAW264.7 cells were transferred to RPMI medium prior to infection and infected at MOI of 100:1. Bacterial RNA was isolated from infected RAW264.7 cells 4 hours post infection using TRIzol and PureLink RNA mini-kit (Invitrogen). cDNA was synthesized using 1 μg of RNA and the High Capacity Reverse Transcription Reagent Kit (Applied Biosystems). Transcripts were quantified using GoTaq qPCR Master Mix (Promega) in a BioRad iQ5 machine. Real-time PCR primers are listed in Additional file 1: Table S4. Relative RNA level of a particular gene in mutant strains was normalized to that of wild type using the 2^−ΔΔ^Ct method with 16S rRNA or *recA* as reference gene [55].

### Mapping transcriptional start sites

The transcriptional start (+1) sites of the promoters were mapped by RNA ligase-mediated rapid amplification of cDNA ends (RLM-RACE) [56]. The RLM-RACE was performed using GeneRacer Kit (Invitrogen) according to manufacturer’s instructions. The *B. pseudomallei* RNA was isolated as described previously [14].

### Sequence motif predication and database search of motifs in the *B. pseudomallei* KHW genome

150 base pairs of nucleotide sequence upstream of the transcriptional starts of each gene was submitted to the bioinformatics tool – MEME (http://meme.nbcr.net/meme/cgi-bin/meme.cgi) for prediction of DNA motifs [57]. The motif with the highest statistical significance (lowest E-value) was chosen and its data – in Position-Specific Probability Matrix format was submitted to MAST (http://meme.nbcr.net/meme/cgi-bin/mast.cgi) to search for the best matching positions in the upstream sequences of *B. pseudomallei* KHW genes [58].

### Statistical analysis

Results were presented as mean ± standard deviation. Student’s t-test was used to find the significant differences between the means, defined as when p < 0.05 (*) and p < 0.01 (**).

## Competing interests

The authors declare no competing interests.

## Authors’ contributions

YC, IS and YHG designed the experiments. YC, IS, CTF, XJY, BET and IJT performed the experiments. YC, IS, CTF, AJ and YHG analyzed the results. YC and YHG conceived the study and together with IS and JFM wrote the manuscript. All authors read and approved the final manuscript.

## Additional file

## Supplementary Material

Additional file 1 **Materials and Methods. Table S1.** Summary of Illumina sequencing. **Table S2.** β-galactosidase activities in *E coli* DH5α strain containing transcriptional promoter-*lacZ* fusions and arabinose-inducible *bsaN* and *bicA* or empty vector. **Table S3.** List of additional plasmids used in this study. **Table S4.** List of Real-Time PCR primers for this study. **Figure S1.** Secretion of BopC. KHW and Δ*bsaM* mutant were grown in acidic LB broth for 3 hours. Total protein from the bacterial culture supernatant was precipitated and protein concentration was normalized with respect to the optical density (OD600) of the bacterial cultures. Proteins on membranes were probed with rabbit polyclonal antibodies to BopC and BopE. **Figure S2.** (A) Intracellular replication of *B. pseudomallei* KHW and mutants in RAW264.7 cells. Cells were infected at an MOI of 0.1:1. Intracellular bacterial loads were quantified at 2 and 8 h post infection by plate counting. (B) Cytotoxicity of *B. pseudomallei* KHW and mutants against RAW264.7 cells. Cells were infected at an MOI of 100:1. Cytotoxicity was quantified at 8 h post infection by LDH release assay. *p < 0.05. **Figure S3.** Secretion and function of BsaN controlled proteins. **A**. Secretion of BPSS1513 in strain KHW. Proteins were separated on 12% polyacrylamide gels, transferred to PVDF membranes and probed with a mouse monoclonal antibody to HA or rabbit polyclonal antibody to BopE. P: pellet; S: supernatant. **B**. Intracellular replication of *B. pseudomallei* KHW and Δ(*BPSS1513-folE)* mutant in RAW264.7 cells at 2 h and 8 h (MOI of 10:1) or **C**. 2 h and 24 h after infection at an MOI of 0.1:1. Intracellular bacterial loads were quantified by plate counting. **D**. Cytotoxicity of *B. pseudomallei* KHW and Δ(*BPSS1513-folE*) mutant against RAW264.7 cells. Cells were infected at an MOI of 100:1. Cytotoxicity was quantified at 8 h post infection by LDH release assay. E. MNGC formation of cells infected with *B. pseudomallei* wild-type (WT) strain KHW and F. Δ(*BPSS1513-1514*) mutant at an MOI of 10:1. Click here for file
