# Supplementary material for: Characterization and analysis of the Burkholderia pseudomallei BsaN virulence regulon
Source: BMC Microbiol. 2014 Aug 1;14:206. doi: 10.1186/s12866-014-0206-6 (PMC4236580; doi:10.1186/s12866-014-0206-6)
Supplement: Additional file 1 — Materials and Methods. Table S1. Summary of Illumina sequencing. Table S2. β-galactosidase activities in E coli DH5α strain containing transcriptional promoter-lacZ fusions and arabinose-inducible bsaN and bicA or empty vector. Table S3. List of additional plasmids used in this study. Table S4. List of Real-Time PCR primers for this study. Figure S1. Secretion of BopC. KHW and ΔbsaM mutant were grown in acidic LB broth for 3 hours. Total protein from the bacterial culture supernatant was precipitated and protein concentration was normalized with respect to the optical density (OD600) of the bacterial cultures. Proteins on membranes were probed with rabbit polyclonal antibodies to BopC and BopE. Figure S2. (A) Intracellular replication of B. pseudomallei KHW and mutants in RAW264.7 cells. Cells were infected at an MOI of 0.1:1. Intracellular bacterial loads were quantified at 2 and 8 h post infection by plate counting. (B) Cytotoxicity of B. pseudomallei KHW and mutants against RAW264.7 cells. Cells were infected at an MOI of 100:1. Cytotoxicity was quantified at 8 h post infection by LDH release assay. *p < 0.05. Figure S3. Secretion and function of BsaN controlled proteins. A. Secretion of BPSS1513 in strain KHW. Proteins were separated on 12% polyacrylamide gels, transferred to PVDF membranes and probed with a mouse monoclonal antibody to HA or rabbit polyclonal antibody to BopE. P: pellet; S: supernatant. B. Intracellular replication of B. pseudomallei KHW and Δ(BPSS1513-folE) mutant in RAW264.7 cells at 2 h and 8 h (MOI of 10:1) or C. 2 h and 24 h after infection at an MOI of 0.1:1. Intracellular bacterial loads were quantified by plate counting. D. Cytotoxicity of B. pseudomallei KHW and Δ(BPSS1513-folE) mutant against RAW264.7 cells. Cells were infected at an MOI of 100:1. Cytotoxicity was quantified at 8 h post infection by LDH release assay. E. MNGC formation of cells infected with B. pseudomallei wild-type (WT) strain KHW and F. Δ(BPSS1513-1514) mutant at a [file s12866-014-0206-6-S1.docx]

**Additional file**

**Materials and Methods**

**Secretion assays**

KHW expressing a C-terminal hemagglutinin (HA) epitope-tagged BPSS1513 construct (pBBR-s1513) was grown in acidic (pH 5.0) LB broth for 3 hours, when bacteria were in their early exponential growth phase. Acidification results in higher T3SS3 expression without impacting cell growth. Following centrifugation and filtration, total protein from the bacterial culture supernatant fraction was precipitated using 10% trichloroacetic acid (TCA). Proteins were separated on 10% polyacrylamide gels, transferred to PVDF membranes and probed with a mouse monoclonal antibody to HA antibody. A polyclonal antibody to BopE was used as positive control.

**Intracellular replication of *B. pseudomallei* in macrophages**

RAW264.7 cells (2.5 x 10^5^ cells/well) were seeded and grown overnight in a 24 well plate. Cells were infected with an MOI of 0.1:1. At 1 hour post infection, infected cells were washed once with PBS, and incubated in fresh culture medium containing kanamycin (250 µg/ml) to kill extracellular bacteria. Infected cells were washed three times with PBS and lysed at 2 and 8 hours post infection with 0.1% (v/v) Triton X-100 and serial dilutions of the lysates were plated onto TSA agar and incubated at 37 °C for 48 hours. Colony counts were then used to calculate bacterial loads.

**Cytotoxicity of *B. pseudomallei* against macrophages**

RAW264.7 cells (2.5 x 10^5^ cells/well) were seeded and grown overnight in a 24 well plate. Cells were infected with an MOI of 100:1. At 1 hour post infection, cells were washed once with PBS, and incubated in fresh culture medium containing kanamycin (250 µg/ml) to kill extracellular bacteria. Cytotoxicity was measured at 8 hours post infection by assaying for lactate dehydrogenase (LDH) release in the cell supernatants using a LDH Cytotoxity Detection Kit (Clontech).

**Multi-nucleated giant cell (MNGC) formation**

RAW264.7 cells (2.5 X 10^5^ cells/well) were seeded and grown overnight in 24 well plates. Cells were infected with *B. pseudomallei* wild-type (WT) strain KHW and *ΔBPSS1513-1514* mutant at an MOI of 10:1. After 2 h of infection, kanamycin (250 µg/ml) was added to the culture medium. At 16 h post infection, cells were washed once with PBS and fixed with 100% methanol (Sigma) for 1 min. Cells were then rinsed once with water and air dried before staining with Giemsa stain for 20 min. After staining, cells were washed once with water and air dried before they were examined for the presence of MNGC under light microscope.

Table S1. Summary of Illumina sequencing

| Strain | Number of non-rRNA reads (millions) | R value  (tech reps) |
| --- | --- | --- |
| KHW-1 | 27.4 | 0.99 |
| KHW-2 | 55.7 |  |
| KHWΔbsaN-1 | 16.1 | 0.99 |
| KHWΔbsaN-2 | 16.8 |  |

Table S2. β-galactosidase activities in *E coli* DH5α strain containing transcriptional promoter-*lacZ* fusions and arabinose-inducible *bsaN* and *bicA* or empty vector

|  | pMLBAD | pMLbsaN/bicA | pMLbsaN | pMLbsaNs/bicA |
| --- | --- | --- | --- | --- |
| PbicA-lacZ | 6.35 ± 0.67 | 127.75 ± 9.71* | 7.29 ± 0.42 | 5.81 ± 0.16 |
| PbopE-lacZ | 6.02 ± 0.12 | 54.69 ± 0.78* | 6.08 ± 0.49 | 4.88 ± 0.02 |
| PbopA-lacZ | 4.29 ± 0.37 | 18.34 ± 1.75* | 5.27 ± 0.20 | 5.83 ± 0.29 |
| PbprA-lacZ | 0.85 ± 0.06 | 1.04 ± 0.03 | ND | ND |
| PbicP-lacZ | 4.84 ± 0.10 | 5.25 ± 0.32 | ND | ND |
| PbprC-lacZ | 4.46 ± 0.27 | 5.24 ± 0.09 | ND | ND |
| PbapA-lacZ | 1.07 ± 0.18 | 1.25 ± 0.12 | ND | ND |

Asterisk (*) indicates statistical significance with p<0.05.

Table S3. List of additional plasmids used in this study

| Plasmid | Relevant characteristic(s)*^a^* | Source or reference |
| --- | --- | --- |
| pMLbsaNs/bicA | pMLBAD containing shorter *bsaN* orf (GTG start) and *bicA* orf from KHW, Tm^r^ | This study |
| pMLbsaN | pMLBAD containing longer *bsaN* orf (ATG start) from KHW, Tm^r^ | This study |
| pRWbprA | PbprA*-lacZ* transcriptional fusion, pRW50mob containing *bprA* upstream sequence from KHW, Tc^r^ | This study |
| pRWbicP | PbicP*-lacZ* transcriptional fusion, pRW50mob containing *bicP* upstream sequence from KHW, Tc^r^ | This study |
| pRWbprC | PbprC*-lacZ* transcriptional fusion, pRW50mob containing *bprC* upstream sequence from KHW, Tc^r^ | This study |
| pRWbapA | PbapA*-lacZ* transcriptional fusion, pRW50mob containing *bapA* upstream sequence from KHW, Tc^r^ | This study |

*^a^* Abbreviations: Km^r^, kanamycin resistant; Tc^r^, tetracycline resistant, Tm^r^, trimethoprim resistant.

Table S4. List of Real-Time PCR primers for this study

| Gene | Sequences (5'-3') |  |
| --- | --- | --- |
| *16S rRNA* | GGCTAGTCTAACCGCAAGGA  TCCGATACGGCTACCTTGTT |  |
| *BPSS1550*  *(bsaJ)* | AAGAACCTGACCGAGCAGCA  TCACCGCATAGCCGGTCTT |  |
| *BPSS1548*  *(bsaL)* | AGGCGAACCTCACGAAAAAC  TTCCGGTACAGGTTGTATTCGG |  |
| *BPSS1547*  *(bsaM)* | TGAAGCTCTGCAACTCCGAATC  AAATACTTCCCCTGACGGAATCG |  |
| *BPSL3302*  *(cheD)* | GGCGGAATGAACCACTTCATG  TTGATCAGCACTTCCATCGC |  |
| *BPSL0280*  *(flgK)* | AACTACAGCGTGTTCCTGTC  GCGACGCCCTTCGATAC |  |
| *BPSL3319*  *(fliC)* | GACGAACTACAACGGCAAGA  ACATGCTTTGCGTGAGGT |  |
| *BPSL3309*  *(motA)* | GACATCTACATGGAGCTGATGG  GATCTTCGGATACTGGGTGAAG |  |
| *BPSS1889* | ATGCGGTGGACGGCTATCATCT  ATCGATACGGAGGACGAACTGCT |  |
| *BPSS0005* | GCTGAAAGTGCTCGAACTGC  TCATCTTATCGCGGAAATGC |  |
| *BPSL0473* | GCACGCCGACCTGTTCTAC  AGGCGACGCAGTTCGATT |  |
| *BPSL0476* | GCCTATGACGAACGCTACGA  ATTCGATCACGTCGAGACAC |  |
| *BPSL0478* | TCAGCGATCTGACGATTCC  GAGGAACACGAGCGTCCA |  |
| *BPSL0484* | GCTCACGATCCATCACACC  AACGTGTGCGTGTCGTAGTC |  |
| *BPSL0487* | ACGTGTGCTGCTCCGATT  GCAGGAGTGGACGACGAAG |  |
| *BPSL0490* | CTACGAATGCGCGAGCTG  TAGCTGAAGCGGTGAATGTC |  |
| *BPSL0492* | CATTTCAAGCCGTTCATGC  GAACCTTCGCGTCCTGTTC |  |
| *BPSL0493* | Gacgaggacgacgaggtt  cgcaagaaggaaatcatca |  |
| *BPSL1793* | AAATTCCCGAACGTGAGGAT  GTCGTTGACGCTGAAGACG |  |
| *BPSL0196* | GCCCTATCAGTGGCACAACA  CTACCACGCCAGTTCACTCC |  |
| *BPSL0197 (metX)* | GCTACCAGGGCGACAAGTTC  CTTCGCCGGATCGAAGTAA |  |
| *BPSL0212 (metK)* | GATCGACACCGTCGTGCT  GAACTTGATGTCGCCCTTGA |  |
| *BPSS1691 (metZ)* | ATTCGGCCACCAAGTTCCT  AACGGGAACACCTTCTCCA |  |

WT Δ*bsaM* WT Δ*bsaM*

55

35

70


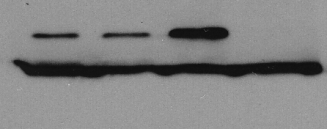

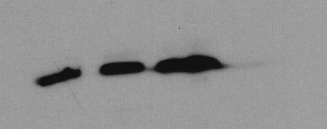


BopC

BopE

Pellet

Supernatant

Fig. S1 Secretion of BopC. KHW and Δ*bsaM* mutant were grown in acidic (pH 5.0) LB broth for 3 hours. Following centrifugation and filtration, total protein from the bacterial culture supernatant fraction was precipitated using 10% trichloroacetic acid (TCA). Protein concentration was normalized with respect to the optical density (OD600) of the bacterial cultures. Proteins were separated on 10% polyacrylamide gels, transferred to PVDF membranes and probed with rabbit polyclonal antibodies to BopC and BopE.

A


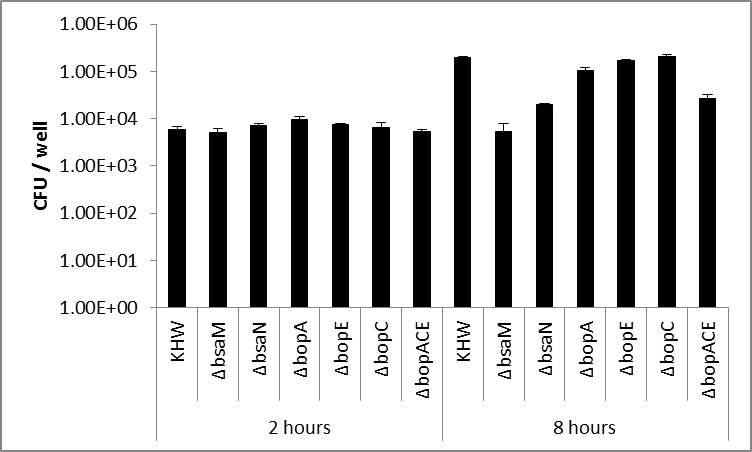


*

*

*

*

B


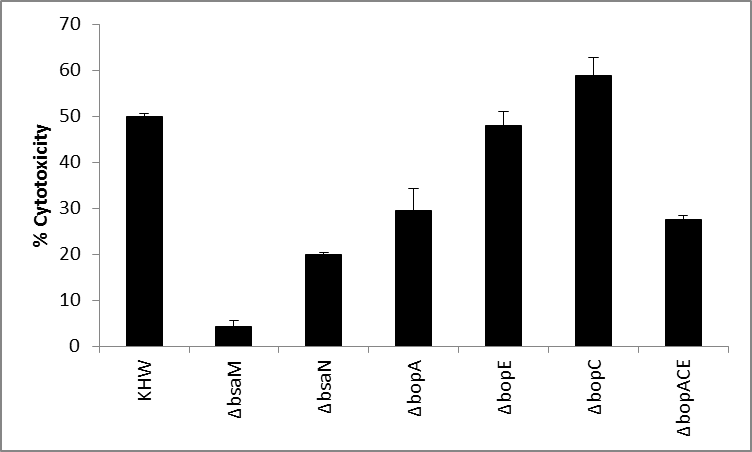


*

*

*

*

Fig. S2 (A) Intracellular replication of *B. pseudomallei* KHW and mutants in RAW264.7 cells. Cells were infected at an MOI of 0.1:1. Intracellular bacterial loads were quantified at 2 and 8 h post infection by plate counting. (B) Cytotoxicity of *B. pseudomallei* KHW and mutants against RAW264.7 cells. Cells were infected at an MOI of 100:1. Cytotoxicity was quantified at 8 h post infection by LDH release assay. *p < 0.05.

A B


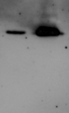

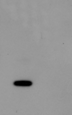


55

35

25

15

10

P

P

S

S

BopE

BPSS1513

C D

E


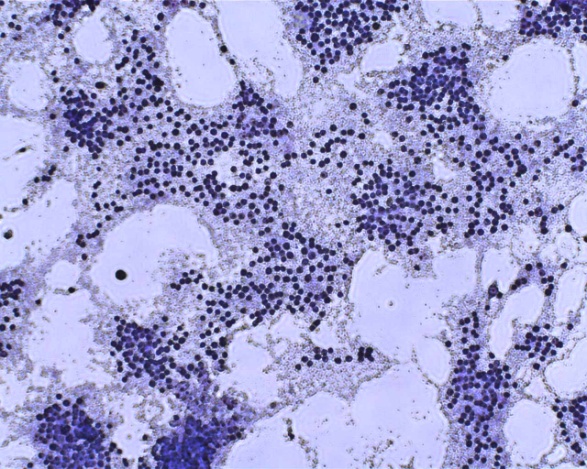

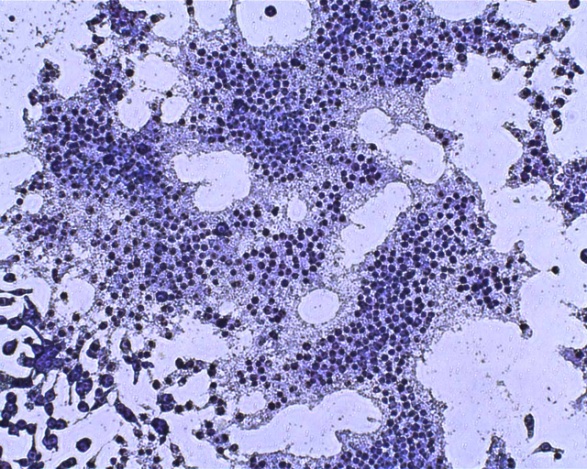


WT

Δ(*BPSS1513-1514)*

Figure S3. Secretion and function of BsaN controlled proteins

**A**. Secretion of BPSS1513 in strain KHW. Proteins were separated on 12% polyacrylamide gels, transferred to PVDF membranes and probed with a mouse monoclonal antibody to HA or rabbit polyclonal antibody to BopE, which serves as a positive control. P: pellet; S: supernatant. **B**. Intracellular replication of *B. pseudomallei* KHW and Δ(*BPSS1513-folE)* mutant in RAW264.7 cells at 2h and 8h (MOI of 10:1) or **C**. 2h and 24h after infection at an MOI of 0.1:1. Intracellular bacterial loads were quantified by plate counting. **D**. Cytotoxicity of *B. pseudomallei* KHW and Δ(*BPSS1513-folE* )mutant against RAW264.7 cells. Cells were infected at an MOI of 100:1. Cytotoxicity was quantified at 8 h post infection by LDH release assay. E. MNGC formation of cells infected with *B. pseudomallei* wild-type (WT) strain KHW and F. Δ(*BPSS1513-1514)* mutant at an MOI of 10:1.
